# Supplementary material for: Combination of GC-MS Molecular Networking and Larvicidal Effect against Aedes aegypti for the Discovery of Bioactive Substances in Commercial Essential Oils
Source: Molecules. 2022 Feb 28;27(5):1588. doi: 10.3390/molecules27051588 (PMC8912102; doi:10.3390/molecules27051588)
Supplement: Supplementary file 1 [file molecules-27-01588-s001.zip › molecules-1591822-supplementary.pdf]

Supplementary Material

Combination of GC-MS Molecular Networking And Larvicidal Effect Against *Aedes Aegypti* for The Discovery of Bioactive Substances in Commercial Essential Oils

Alan Cesar Pilon[1,†], Marcelo Del Grande[1,†], Maíra R. S. Silvério[1], Ricardo R. Silva[1], Lorena C. Albernaz[2], Paulo Cesar Vieira[1], João Luis Callegari Lopes[1], Laila S. Espíndola[2], Norberto Peporine Lopes[1,*]

1 NPPNS, Departamento de Ciências Biomoleculares, Faculdade de Ciências Farmacêuticas de Ribeirão Preto, Universidade de São Paulo -USP, Ribeirão Preto, São Paulo, Brazil.

2 Universidade de Brasília, Laboratório de Farmacognosia, Campus Universitário Darcy Ribeiro, 70910-900, Brasília, DF, Brazil.

* Correspondence: npelopes@fcfrp.usp.br

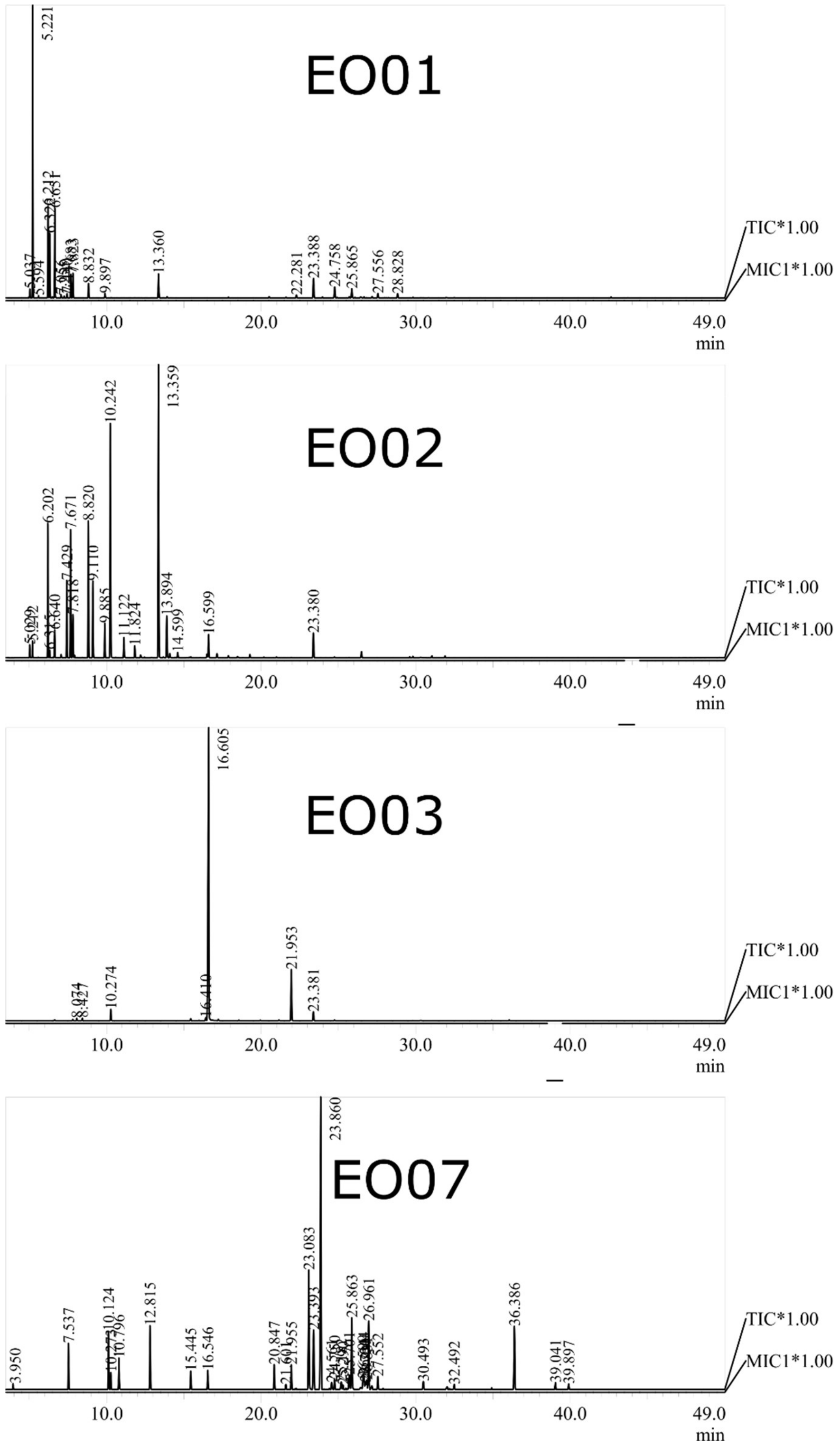

Figure S1. GC-EI-MS chromatograms of commercial essential oils with high larvicidal activity against *Ae. aegypti* larvae. *Juniperus communis* (Cupressaceae) EO01; *Origanum majorana* (Lamiaceae) EO02; *Cymbopogon martini* (Poaceae) EO03 and *Boswellia carteri* (Burseraceae) EO07.

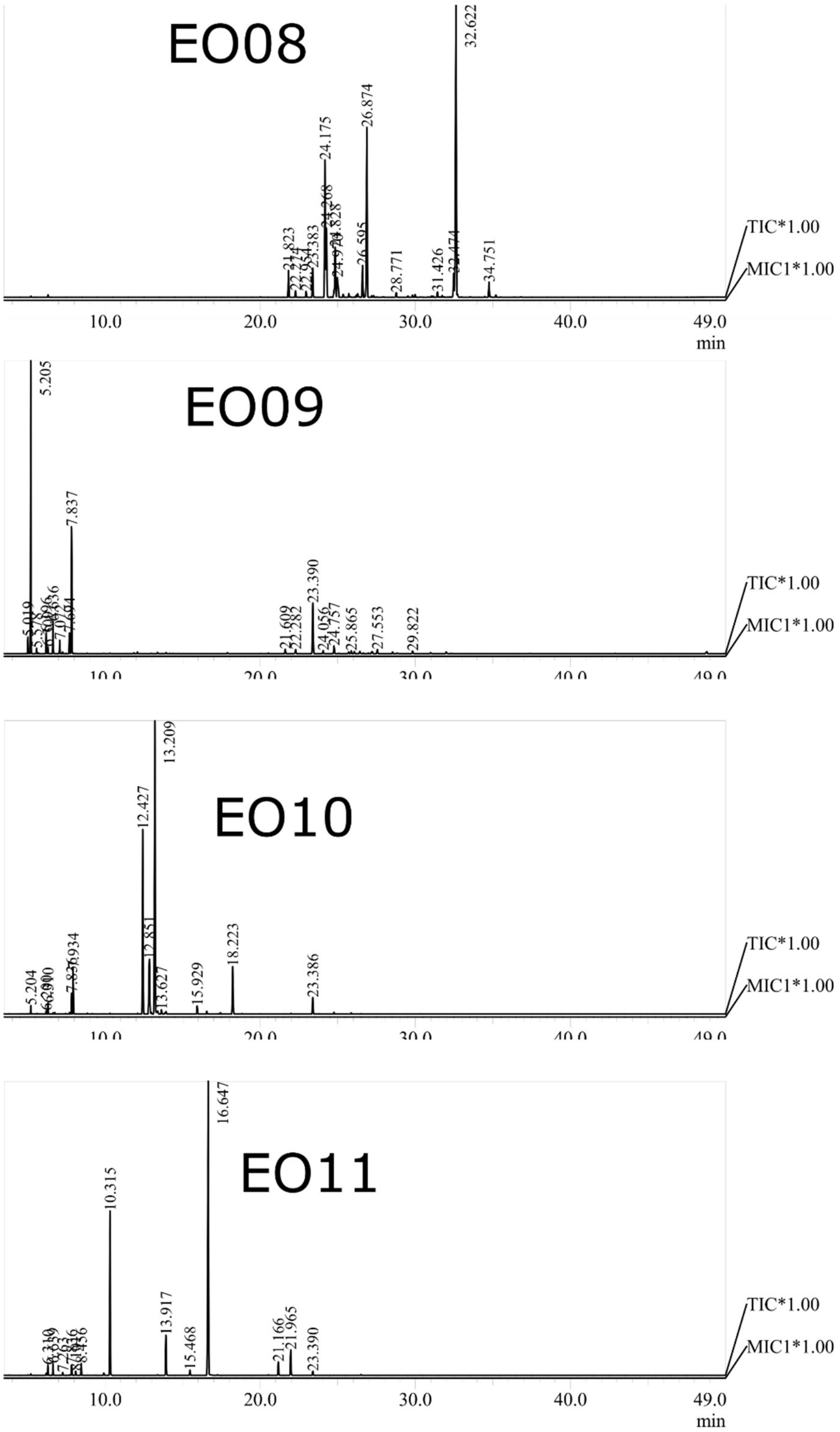

Figure S2. GC-EI-MS chromatograms of commercial essential oils with high larvicidal activity against *Ae. aegypti* larvae. *Mentha piperita* (Lamiaceae) EO08; *Citrus aurantium var. amara* (Rutaceae) EO09; *Eucalyptus citriodora* (Myrtaceae) EO10 and *Eucalyptus globulus* (Myrtaceae) EO11.

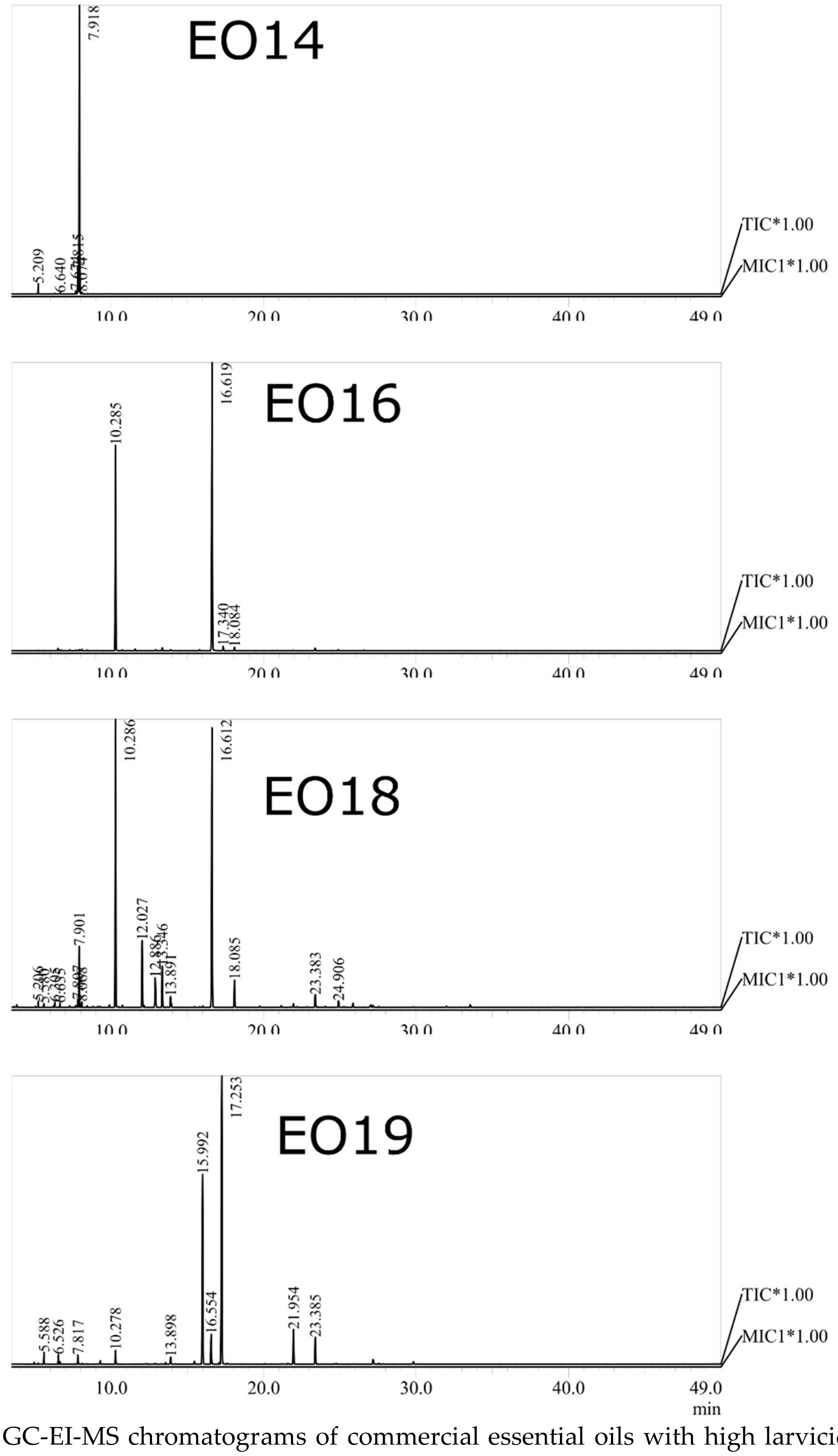

Figure S3. GC-EI-MS chromatograms of commercial essential oils with high larvicidal activity against *Ae. aegypti* larvae. *Lavandula angustifolia* (Lamiaceae) EO14; *Lavandula hybrida* (Lamiaceae) EO16; *Cymbopogon flexuosus* (Poaceae) EO18 and *Cymbopogon nardus* (Poaceae) EO19.

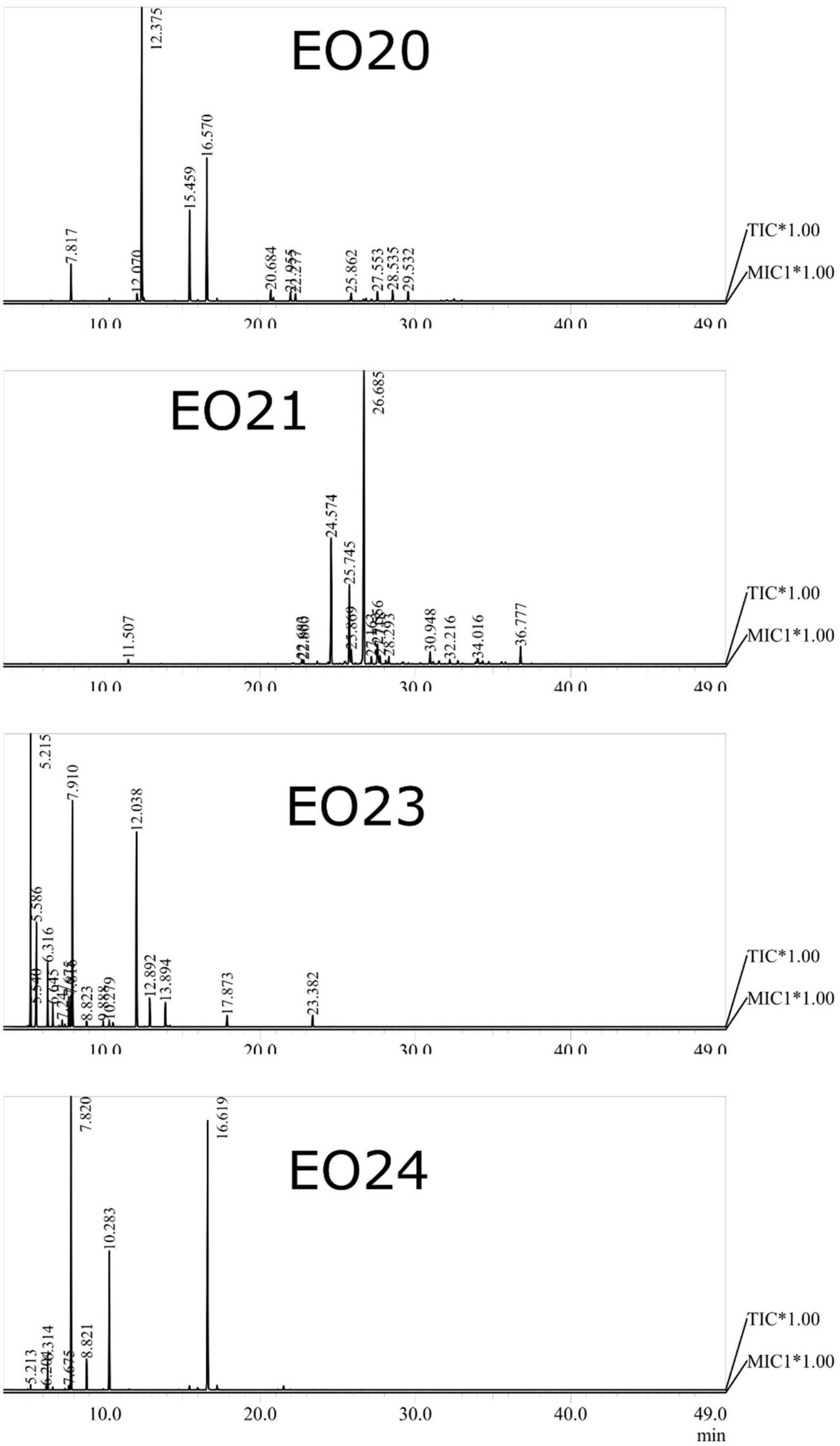

Figure S4. GC-EI-MS chromatograms of commercial essential oils with high larvicidal activity against *Ae. aegypti* larvae. *Cedrus atlantica* (Pinaceae) EO20; *Rosmarinus officinalis* (Lamiaceae) EO21; *Citrus aurantium subsp. Bergamia* (Rutaceae) EO23 and *Perlagonium graveolens* (Geraniaceae) EO24.

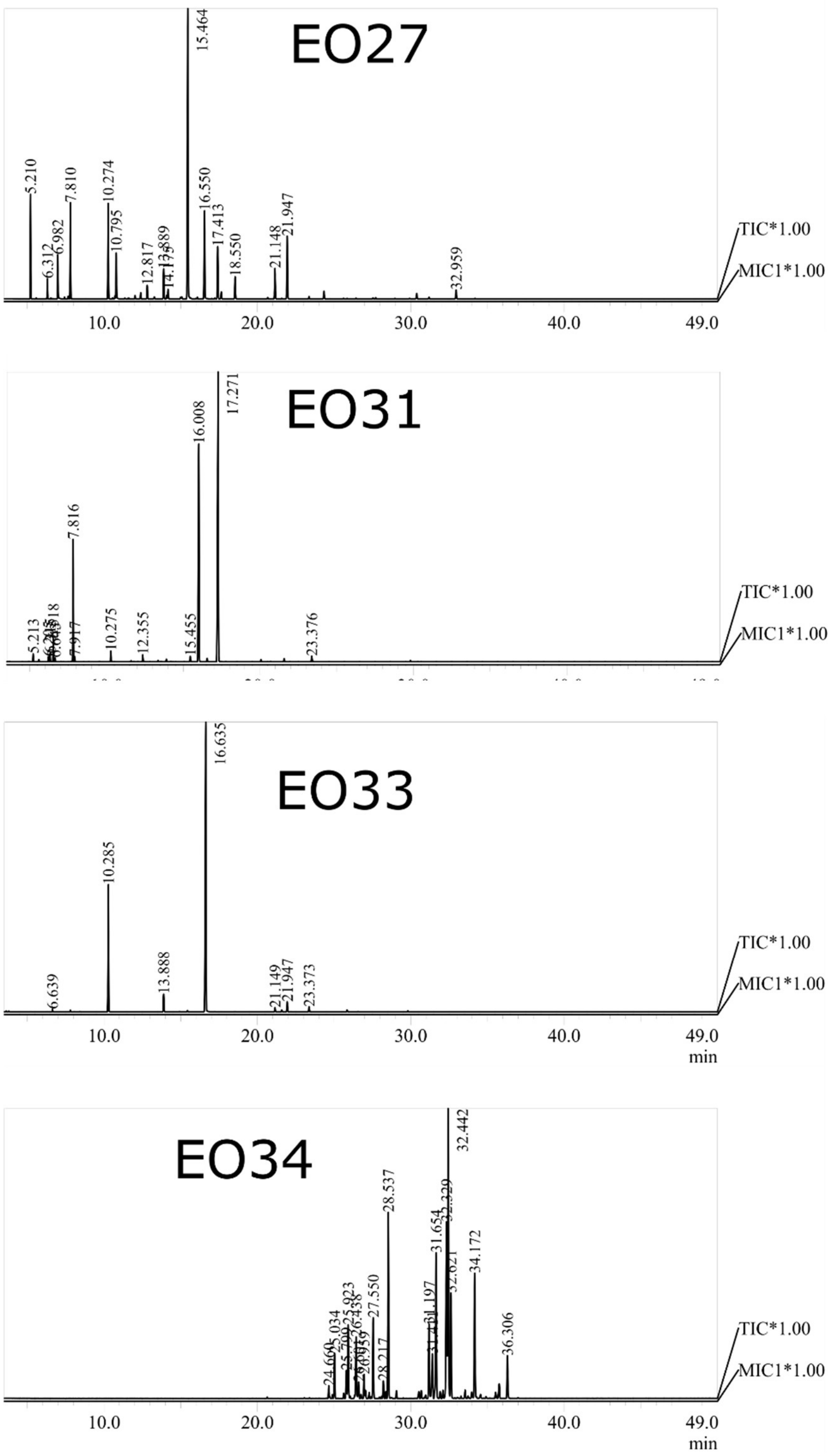

Figure S5. GC-EI-MS chromatograms of commercial essential oils with high larvicidal activity against *Ae. aegypti* larvae. *Litsea cubeba* (Lauraceae) EO27; *Salvia sclareia* (Lamiaceae) EO31; *Amyris balsamifera* (Rutaceae) EO33 and *Eucalyptus staigeriana* (Myrtaceae) EO34.
